# Operating Properties of Deep Hole Boring Tools with Modified Design

**DOI:** 10.3390/ma17071551

**Published:** 2024-03-28

**Authors:** Norbert Kępczak, Grzegorz Bechciński, Radosław Rosik

**Affiliations:** Institute of Machine Tools and Production Engineering, Faculty of Mechanical Engineering, Lodz University of Technology, Stefanowskiego 1/15, 90-537 Lodz, Poland; grzegorz.bechcinski@p.lodz.pl (G.B.); radoslaw.rosik@p.lodz.pl (R.R.)

**Keywords:** boring bar, modal analysis, finite element method, dynamic properties, static properties

## Abstract

This paper presents the results of research work on the revised design of a deep hole boring tool. The study was divided into three stages: theoretical, experimental and operational. In the theoretical part, a 3D model of the actual boring bar was created, which was subjected to strength tests using the Finite Element Method (FEM), and then prototypes of new deep hole boring tools were made with structural modifications to the shank part of the tool. For the polymer concrete core of a shank, there was a 14.59% lower displacement, and for the rubber-doped polymer concrete (SBR—styrene butadiene rubber) core of a shank there was a 4.84% lower displacement in comparison to the original boring bar. In the experimental part of the study, the original boring bar and the prototypes were subjected to experimental modal analysis and static analysis tests to compare dynamic and static properties. In the operational part of the study, boring tests were carried out for various workpiece materials, during which the basic parameters of the surface geometric structure (SGS), such as roughness Ra and Rz, were studied. Despite the promising preliminary results of the theoretical and experimental studies, using the described modifications to the boring bar is not recommended.

## 1. Introduction

Vibrations have a negative effect on the quality of the machined surface and can damage the machine tool or the tool. Vibrations occurring during machining of the workpiece material can be divided into forced and self-excited [1,2]. Forced vibration occurs as a result of an external impulse or periodically acting excitation force. Self-excited vibration, unlike forced vibration, is not caused by an external disturbance but by a dynamic interaction between the mechanical system and the machining process [3,4,5].

In the optimization of deep hole boring processes, monitoring the condition of the surface layer of the workpiece plays an important role in effective tool wear replacement policy, product quality control and lower production-related costs. The work of Xiao et al. [6] proposes a novel approach to monitoring the condition of the workpiece surface layer using deep boring based on the second-generation wavelet transform.

Other works describe the topic of boring deep holes using a cylindrical countersink with a laser system for monitoring the condition of the workpiece surface layer instead of a conventional boring bar. The study by Katsuki et al. [7] describes improvements to three main aspects of laser tooling: a method of applying voltage to the piezoelectric actuators used to control tool position and inclination, the speed of the actuator response, and the strategy of the conductor.

Khoroshailo et al. [8] attempt to build a tooling system that effectively dampens vibrations during deep hole boring. The paper presents a mathematical model of the vibration of the machining tip under the influence of variable forces, which has been applied to a novel tooling. A three-dimensional model was also created, based on which the tooling design was developed. As a result, machining tip displacement diagrams were obtained to evaluate the decrease in vibration amplitude values during the boring process. Experimental tests conducted showed increased vibration resistance of boring tools using the developed tooling system [8].

It is important to accurately determine the values of the frequencies at which the resonance phenomenon occurs. Nowadays, numerical analyses can be performed at the design stage, as a result of which the designer obtains the forms of vibrations and their frequencies [9]. However, a numerical model is always an ideal model in which there are no defects. To confirm the results obtained theoretically, it is necessary to carry out a so-called identification experiment, which allows full verification of the numerical model.

This article presents the results of work on the revised design of the deep hole boring tool. The study was divided into three parts: theoretical, experimental and operational. In the theoretical part, a three-dimensional model of the actual boring tool was created, on the basis of which new deep hole boring tools were then prototyped with a changed geometry of the shank part of the tool. This change consisted of making a hole in the shank part of the tool and filling it with various construction materials. With the help of static analysis and parameterization of the structure, two prototypes were indicated, which were manufactured and subjected to further experimental and operational tests. In the experimental part of the study, the original boring bar and the prototypes were subjected to modal analysis and static analysis tests to compare dynamic and static properties. In the operational part of the study, boring tests were carried out for various workpiece materials, during which the basic parameters of the surface geometric structure (SGS), which are the roughness Ra and Rz, were examined. Novel to the research was the application of different materials with a high dumping ratio as a core for the shank part of the boring bar in order to improve the dynamic properties of the boring bar and reduce vibrations during the cutting process.

## 2. Methods

**Figure 1:**
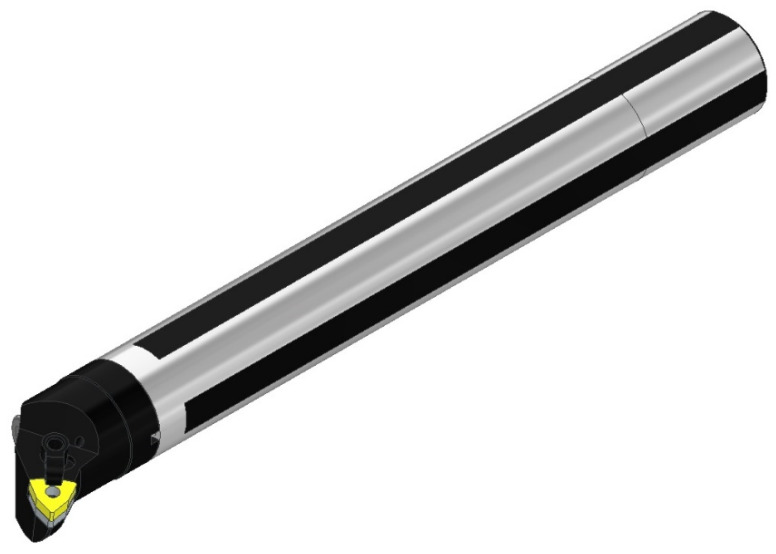
Three-dimensional model of PAFANA’s boring bar.

PAFANA’s Smart Head System boring bar was used in the study. This is a modular tool which includes a head with the designation K40-MWLNR/L08 (Pabianicka Fabryka Narzędzi “PAFANA” S.A., Pabianice, Poland) and a shank with the designation A40-K40 300 (Pabianicka Fabryka Narzędzi “PAFANA” S.A., Pabianice, Poland) [10]. At the first stage of the research, the experimental verification of the deep hole boring bar model was performed [11]. A 3D model of the boring bar is shown in Figure 1.

**Figure 2:**
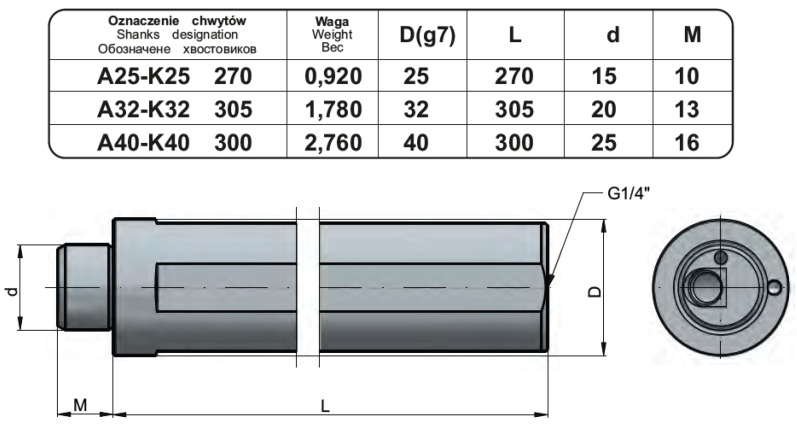
Dimensions of the shank portion of the boring bar [10].

In connection with the planned modification of the shank part of the boring bar, Figure 2 shows the basic dimensions of the design element.

Based on the results of the theoretical research, prototypes of two boring bars with a modified shank section were produced in the experimental part. The material used for the modification was Epument 140/5 A1 polymer concrete, offered by RAMPF (RAMPF Machine Systems GmbH & Co. KG, Wangen bei Göppingen, Germany). The manufacturer provides all the components for making the mineral cast yourself. The kit includes three components: epoxy resin, hardener and aggregate mixture. The manufacturer includes how to prepare the material and the dedicated mixture ratio, which is the following: 2.2 (epoxy resin): 0.6 (hardener): 27.2 (aggregates) [12]. Polymer concrete (PC) also goes by the name of mineral cast. It is a composite material consisting of inorganic aggregates such as basalt, spodumene (LiAlSi_2_O_6_), fly ash, river gravel, sand, chalk, etc., bonded together with resin [13,14,15]. The most commonly used resins are epoxy [13], polyester [14] and vinylester resins [15].

In order to determine the dynamic properties of the tool, experimental modal analysis was used in the study. Experimental modal analysis is a frequently used technique for studying the dynamic properties of mechanical objects, both at the design stage and in the operation of machinery. The identification experiment in the experimental modal analysis involves forcing an object to vibrate while measuring the forcing force and the response of the system, usually in the form of a spectrum of vibration acceleration [16,17,18].

In order to confirm the results of the theoretical and experimental studies in the operational part of the research, boring tests were carried out for various machining materials (steel 18G2A and aluminum PA4), during which the basic parameters of the surface geometric structure (SGS), which are the roughness Ra and Rz, were studied. During the boring tests, three machining parameters were changed in the following ranges:

- Cutting speed *v_c_* = 19 ÷ 271 m/min;
- Feed rate *f* = 0.1 ÷ 1 mm/rev;
- Depth of cut *a_p_* = 0.5 ÷ 2 mm.

## 3. Results and Discussion

### 3.1. Theoretical Study

The Finite Element Method (FEM) is a common simulation technique used to test existing structures under various boundary conditions. Very often, FEM is also used at the design and prototyping stage to determine basic static and dynamic properties [19].

**Figure 3:**
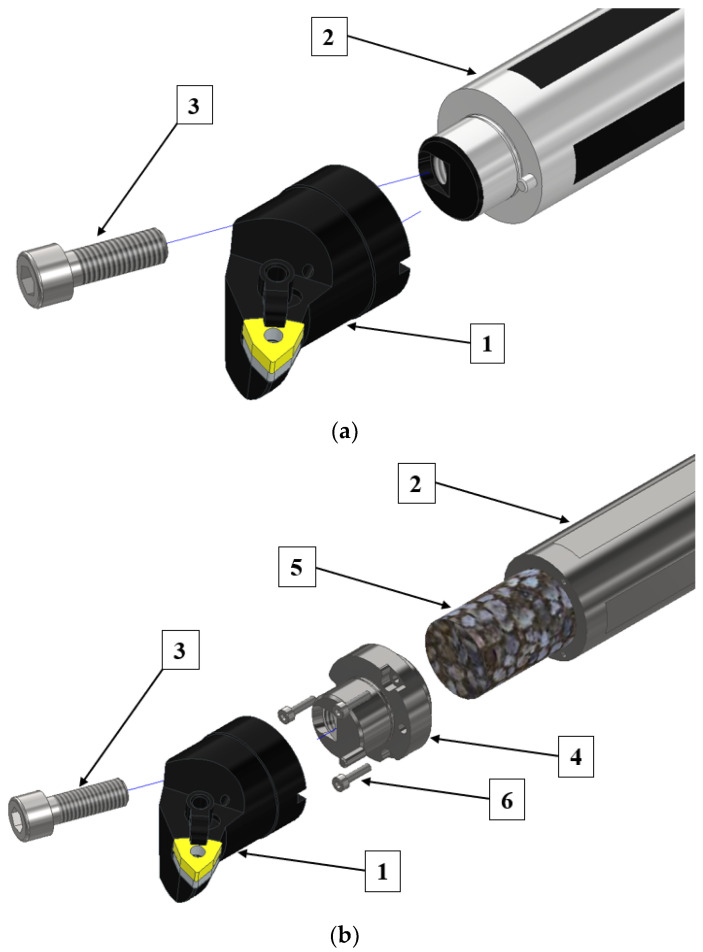
Comparison of the original design (**a**) with the modified design (**b**). 1—head, 2—shank, 3—fastening screw M10x30, 4—fastener, 5—filler core, 6—fastener fixing screws.

The theoretical part involved carrying out a modification of the shank part of the boring bar in such a way as to obtain a hole which was then filled with various structural materials to increase the rigidity of the entire boring bar. To this end, the design of the boring bar was modified first. Figure 3 shows a comparison between the original design and the modified design.

The original boring bar (Figure 3a) consists of three elements: head (1), shank (2) and M10x30 fastening screw (3). The modified design (Figure 3b) consists of six components: head (1), shank (2), M10x30 fastening screw (3), fastener (4), filler core (5) and three M3x12 fastening screws for the fastener (6).

**Figure 4:**
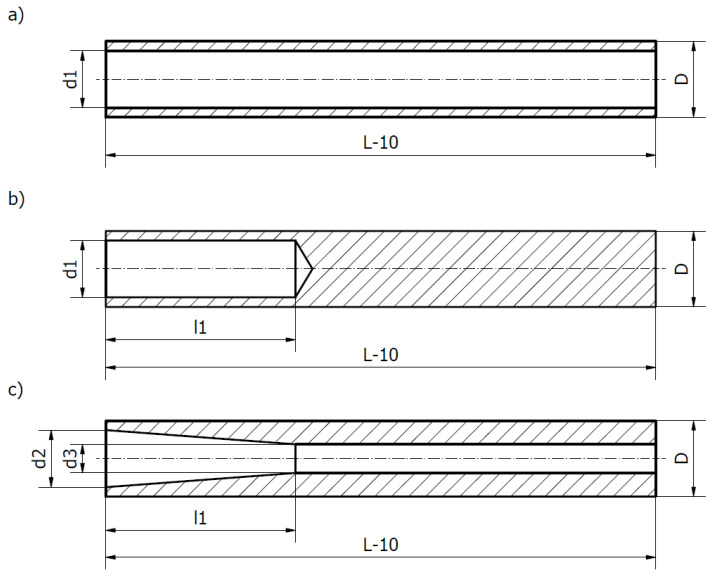
Schematic representation of proposed shank holes for filler core. (**a**) Through hole, (**b**) blind hole, (**c**) tapered hole.

Numerical tests were carried out in Autodesk Inventor Professional. At the initial stage, the effect of core shape and core filler material on the displacement of the tip of a boring bar loaded with an example peripheral force of 300 N was studied. Figure 4 shows the types of core holes that were made in the shank part of the boring bar and subjected to numerical tests. Through holes with a variable internal diameter value (Figure 4a), blind holes with a variable internal diameter values and variable depth values (Figure 4b), as well as tapered holes with variable front and back internal diameter values and variable depth values (Figure 4c) were considered. Table 1 shows the values of the variable parameters.

**Figure 5:**
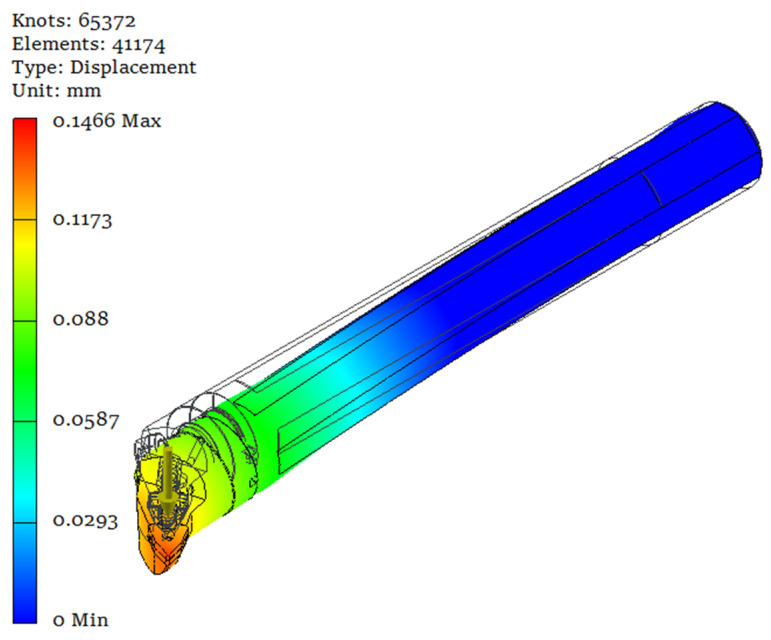
Results of static analysis of PAFANA’s boring bar.

These holes were filled with various structural materials to form cores. A material with a higher density than the steel from which the shank was made (the lead) was considered in order to increase the weight of the boring bar and thus its stiffness, as well as materials with very good vibration dampening properties (rubber, polymer concrete, polymer concrete doped with rubber and resin). Cast iron was not considered, as it would have been a very complicated process to make a core casting in a steel shank. A preliminary numerical study demonstrated that the smallest displacement values were obtained for solutions with holes of a fixed diameter value of Φ30 × 200 mm when filled with polymer concrete and rubber-doped polymer concrete material. Figure 5 shows the results of the static analysis for the original PAFANA boring bar, while Table 2 summarizes the comparative results of the analyses for different core materials.

As can be seen from the figure and the table above, a displacement value of 0.1466 mm was obtained for the original PAFANA boring bar. Only two of the materials considered obtained a displacement value lower than the original design (polymer concrete and rubber-doped polymer concrete (SBR)), and these materials were used in further considerations. For the polymer concrete core of a shank, there was 14.59% lower displacement, and for the rubber-doped polymer concrete (SBR—styrene butadiene rubber) core of the shank there was a 4.84% lower displacement in comparison to the original boring bar.

Autodesk Inventor Professional has a built-in module that allows one to perform multi-parameter parametric analysis. In the following part of the numerical study, a two-parameter optimization of the shank bore for the filler core was performed to determine the best combination of diameter values and bore length, for which the value of the displacement of the machining tip of the boring bar would be smaller than the displacement of the machining tip of the original boring bar, and for which the lowest mass of the entire boring bar was obtained. Table 3 presents a summary of results for the prototype boring bar with a core made of polymer concrete (PC), while Table 4 presents a summary of results for a prototype boring bar with a core made of polymer concrete doped with rubber (PC + SBR).

As can be seen from Table 3, for a PC core, the lowest values of displacement/mass were obtained for combinations d1/l1 as Φ20 × 200 mm and 30 × 283 mm (marked in brown). The most optimal combination indicated by Autodesk Inventor was Φ25 × 200 mm (marked in green) when the displacements marked in red were greater than PAFANA’s original boring bar. As can be seen from Table 4, for the PC + SBR core, the lowest values of displacement/mass were obtained for combinations d1/l1 as Φ30 × 200 mm and 30 × 283 mm (marked in brown). The most optimal combination indicated by Autodesk Inventor was Φ30 × 200 mm (marked in green) when the displacements marked in red were greater than PAFANA’s original boring bar.

Taking into account that during the manufacture of the actual prototypes of both boring bars the fastener fixing screws are screwed directly into the wall of the shank, for technological reasons, and in order to ensure the proper connection of the two elements, a combination diameter and length of Φ25 × 200 mm was selected for further consideration.

### 3.2. Experimental Study

**Figure 6:**
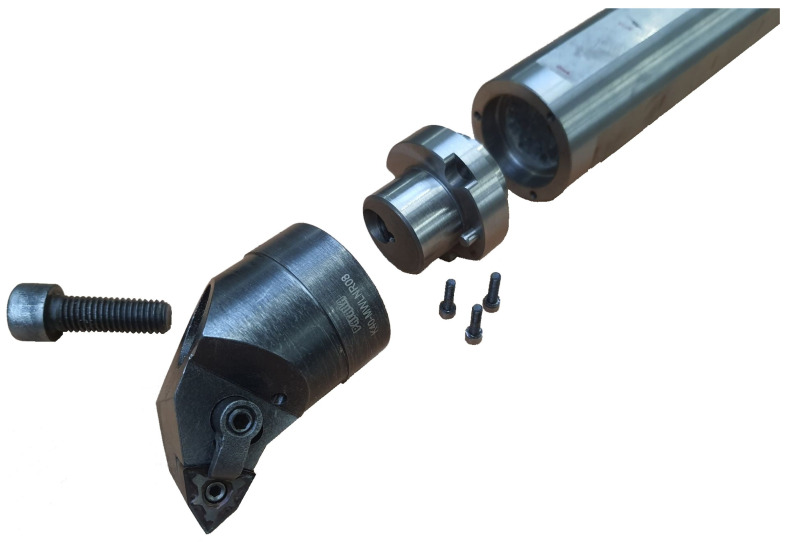
View of the prototype boring bar.

The experimental study was divided into two parts. In the first part, an experimental modal analysis was conducted to determine the dynamic properties of the prototype boring bars compared to the original boring bar. In the second part, static tests were conducted to determine the value of displacement depending on the applied load. Figure 6 shows a view of the prototype boring bar with polymer concrete filling.

#### 3.2.1. Dynamic Properties

An experimental modal analysis was carried out to determine the dynamic properties of the prototype boring bars. This analysis is a frequently used technique for studying the dynamic properties of mechanical objects, both at the design stage and in the operation of machines. Unlike operational modal analysis [20], an identification experiment involves forcing an object to vibrate while measuring the forcing force and the response of the system, usually in the form of a spectrum of vibration accelerations [18,21,22,23].

**Figure 7:**
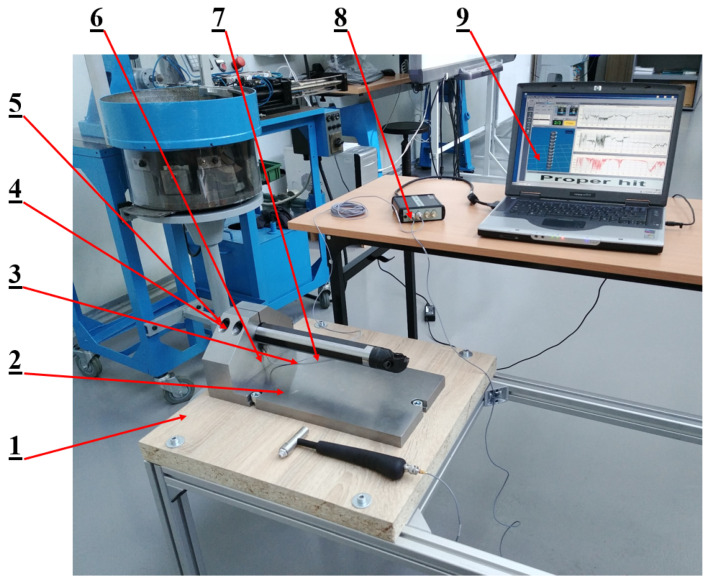
Actual test stand. 1—frame, 2—modal hammer, 3—base, 4—support, 5—mounting screws, 6—accelerometer, 7—boring bar, 8—data acquisition module, 9—computer [11].

Due to hardware limitations, a SISO (Single Input Single Output) procedure was used in this study. Figure 7 shows the actual test stand with the apparatus for conducting experimental modal analysis, which includes the following: (1) frame, (2) modal hammer, (3) base, (4) support, (5) mounting screws, (6) accelerometer (7) boring bar, (8) data acquisition module, and (9) computer with software.

The PULSE Lite system from Brüel and Kjær was used for measurement and data acquisition, which includes the following:

- Accelerometer 4514 [24].
- Modal hammer 8206-003 [25].
- 3560-L data acquisition module.

The “modal assistant” of the PULSE LabShop software version 12.5.0.196 allows one to perform a fast Fourier transform (FFF) of the collected data.

**Figure 8:**
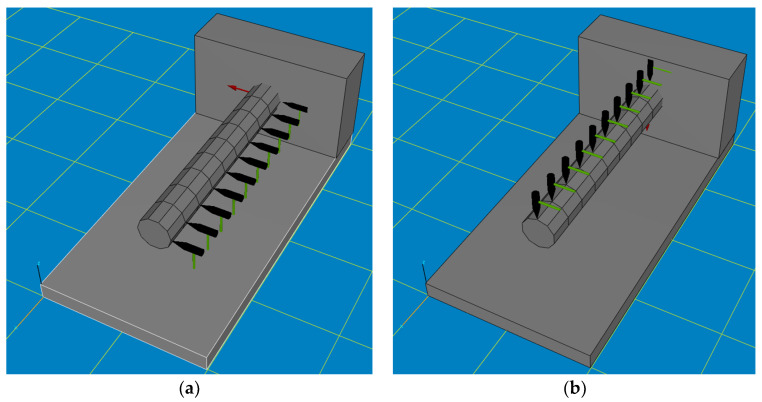
Approximate geometric model of the test stand. (**a**) Location of points excited to vibration in the horizontal direction, as well as the location of attachment of the displacement sensor. (**b**) Location of points excited to vibration in the vertical direction as well as the location of attachment of the displacement sensor.

After the settings were made in the program, the shape of the test object had to be defined in the Pulse Lite software version 12.5.0.196 (Figure 8), and the forcing locations (green-black hammers) and the accelerometer mounting location (red arrow) had to be indicated. Due to the limited spatial modeling capabilities of the Pulse Lite software, the shape of the actual boring bar was modeled roughly as a cylinder. On the boring bar, 10 measuring points were determined in both the vertical and horizontal directions, which were sequentially excited three times to vibration. The accelerometer was placed on the boring bar as close to the bracket as possible. The boring bar was tested three times. The test was conducted in the frequency domain in a range from 0 to 3200 Hz. The sampling rate was 6400 Hz, while the recorded signal time was 1 s.

**Figure 9:**
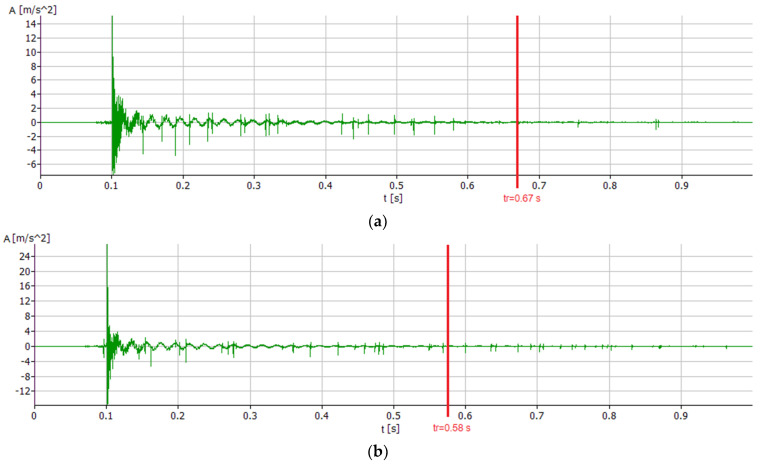

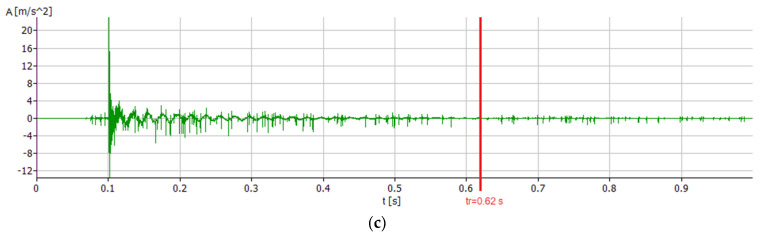
Time course of impulse excitations in the vertical direction (**a**) for the original PAFANA boring bar, (**b**) for the PC boring bar and (**c**) for the PC + SBR boring bar.

**Figure 10:**
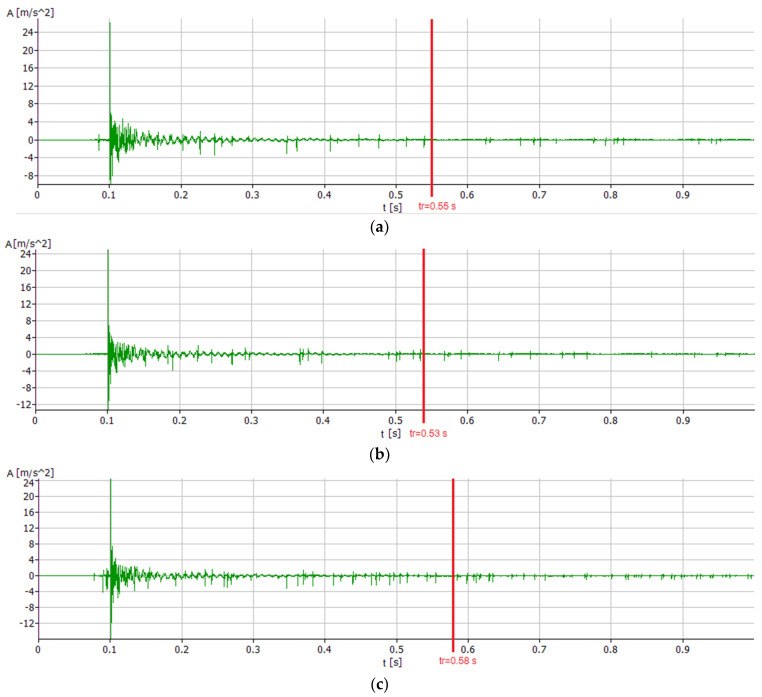
Time course of impulse excitations in the horizontal direction (**a**) for the original PAFANA boring bar, (**b**) for the PC boring bar and (**c**) for PC + SBR boring bar.

First, the time courses of the system’s response to a single impulse forcing were analyzed, as shown in Figure 9 and Figure 10.

All courses are characterized by the fact that immediately after excitation to vibration the system behaves very chaotically, while approximately after time *t* = 0.02 s the courses arrange themselves into clear pulsating waves from which the free vibration frequencies of the individual modes, as well as the damping coefficients, were calculated.

The analysis of the courses also shows that the PC boring bar has the shortest relaxation time of *t_r_* = 0.58 s for the vertical direction and *t_r_* = 0.53 s for the horizontal direction. The relaxation time of the PC + SBR boring bar was *t_r_* = 0.62 s for the vertical direction and *t_r_* = 0.58 s for the horizontal direction, respectively. In the horizontal direction, this was the longest relaxation time. The longest relaxation time for the vertical direction was noted for the original PAFANA boring bar, in which *t_r_* = 0.67 s and *t_r_* = 0.55 s were obtained for the horizontal direction.

**Figure 11:**
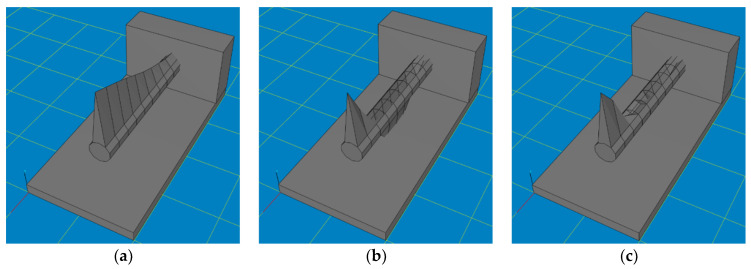
Obtained forms of free vibration in the vertical direction. (**a**) First mode, (**b**) second mode, (**c**) third mode.

**Figure 12:**
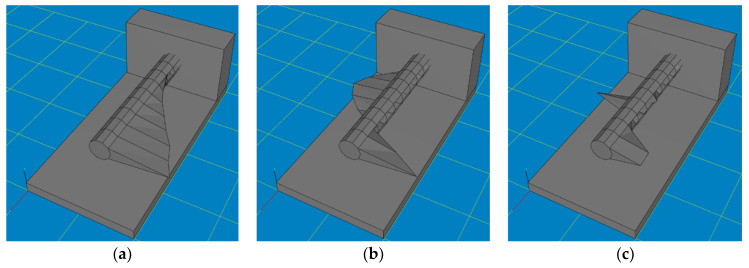
Obtained forms of free vibration in the horizontal direction. (**a**) First mode, (**b**) second mode, (**c**) third mode.

The frequency courses of the H1 transition function were then analyzed. Figure 11 and Figure 12 show the free vibration modes that were identified for the original PAFANA boring bar. In addition, Table 5 and Table 6 present the values of the free vibration frequencies of the different modes, the amplitude of the H1 transition function estimate, as well as the vibration damping coefficients.

As can be seen from Table 5 for the vertical direction, for the first mode of free vibration there was a slight increase in the frequency values for both the PC boring bar (266.00 Hz) and the PC + SBR (265.67 Hz) compared to the original boring bar (260.00 Hz). For both prototype boring bars, there was a decrease in the amplitude value of the transition function estimate by 13.0% and 2.0%, respectively, and an increase in the damping ratio value by 37.9% and 31.8%, respectively. The second mode of free vibration was defined only for the original PAFANA boring bar and the prototype PC boring bar. In this case, there was a 2.8% decrease in vibration frequency values, a 76.3% decrease in amplitude values and a 61.9% increase in the damping ratio. For the PC + SBR tool, the second mode of vibration could not be defined. The third mode of free vibration was defined only for the original PAFANA boring bar.

As can be seen from Table 6 for the horizontal direction, for the first mode of free vibration, there was also an increase in frequency values for both the PC boring bar (343.67 Hz) and the PC + SBR (345.33 Hz) compared to the original boring bar (314.33 Hz). For both prototype boring bars, there was an increase in the amplitude value of the transition function estimate by 16.6% and 17.1%, respectively, and a decrease in the damping ratio value by 6.2% and 7.3%, respectively. The second mode of free vibration was defined only for the original PAFANA boring bar and the prototype PC boring bar. In this case, there was a 2.2% decrease in the value of the vibration frequency, a 53.3% decrease in the value of the amplitude and a 92.7% increase in the damping ratio. For the PC + SBR tool, the second mode of vibration could not be defined. The third mode of free vibration was defined only for the original PAFANA boring bar.

The lack of disclosure of the third mode for the PC boring bar and the second and third modes for the PC + SBR boring bar may be due to the use of a filler material of polymer concrete and polymer concrete doped with rubber granules, where both materials have a very high vibration damping ability.

Despite the observation of one case where the dynamic properties of both prototype boring bars decreased compared to the original boring bar, an overall increase in the dynamic properties of PC and PC + SBR boring bars was found with respect to the PAFANA boring bar.

#### 3.2.2. Static Properties

**Figure 13:**
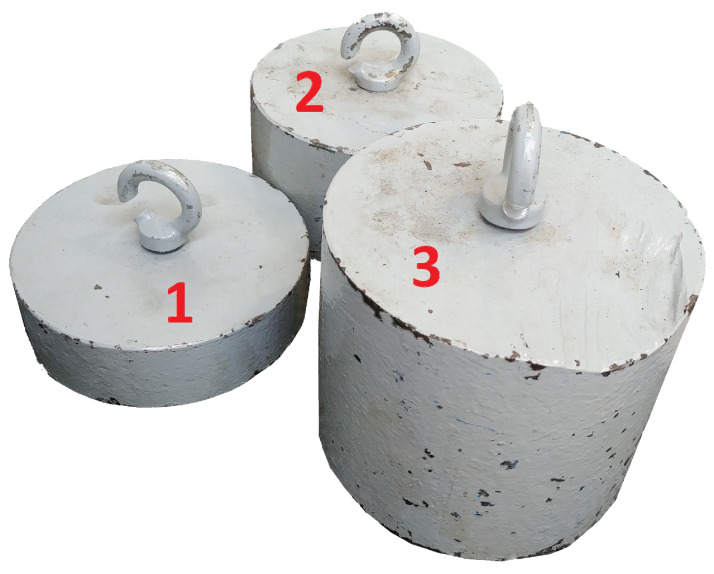
View of the prepared weights with the following masses: 1—10 kg, 2—20 kg, 3—30 kg [11].

In order to conduct experimental tests of the static properties of the boring bar on the test stand, three weights were prepared, with which it was loaded accordingly. Figure 13 shows a view of the prepared weights.

**Figure 14:**
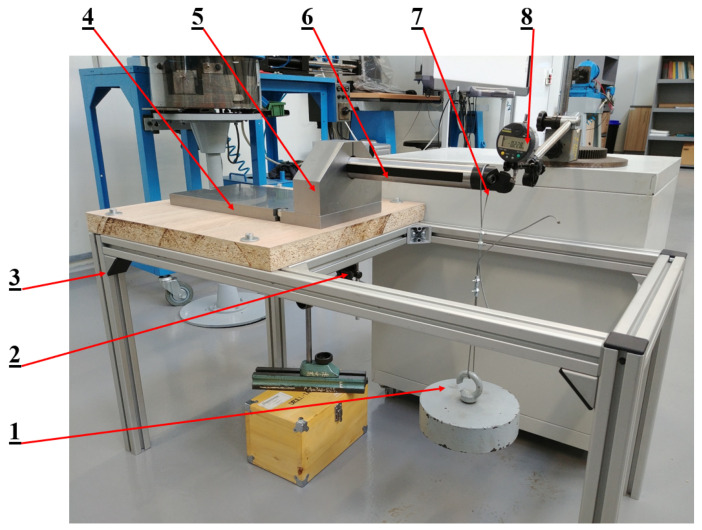
View of the test stand. 1—mass, 2—bottom sensor, 3—frame, 4—base, 5—support, 6—boring shank, 7—boring head, 8—top sensor [11].

The boring bars were then mounted successively on the test stand and loaded accordingly. The differential displacement of the boring head was measured using two displacement sensors. One of the sensors was placed directly over the boring head (top sensor), while the other was placed under the frame of the test stand (bottom sensor). Figure 14 shows a view of the test stand consisting of the following: (1) weight, (2) displacement sensor under the test stand frame, (3) frame, (4) base, (5) bracket, (6) boring shank, (7) boring head and (8) boring head displacement sensor.

**Figure 15:**
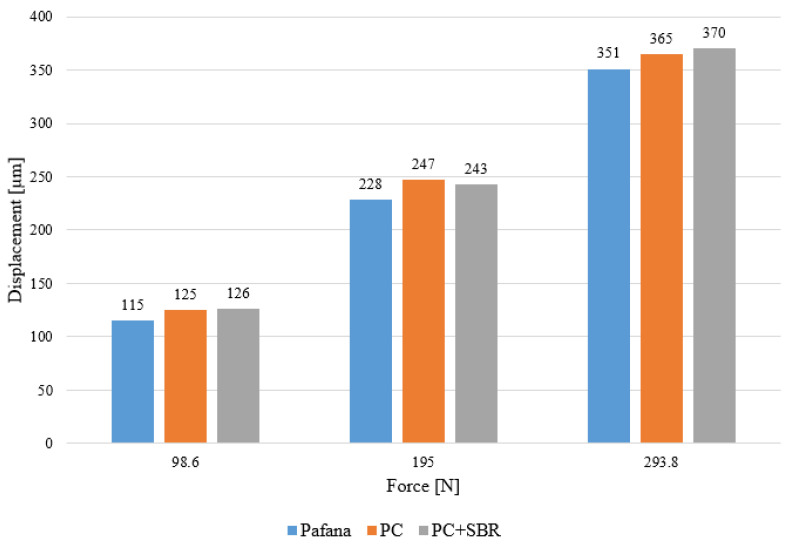
Comparison of results of displacement of all boring bars under different loads.

Table 7, Table 8 and Table 9 present the obtained displacement results for each load. Figure 15 shows a comparison of the results of displacement measurements for all boring bars.

From the above tables and figure, it is clear that the most rigid tool is the original PAFANA boring bar, as it obtained the smallest displacements for all load tests.

#### 3.2.3. Operational Properties

In order to verify the results of the theoretical and experimental studies, operational tests were carried out. Boring tests were carried out for two different machined materials (steel 18G2A and aluminum PA4). The authors decided to select commonly machined materials to observe phenomena that can occur during machining traditional materials. During tests, basic parameters of the surface geometric structure (SGS), such as roughness Ra and Rz, were studied. During the boring tests, three machining parameters were changed in the following ranges:

- Cutting speed *v_c_* = 19 ÷ 271 m/min;
- Feed rate *f* = 0.1 ÷ 1 mm/rev;
- Depth of cut *a_p_* = 0.5 ÷ 2 mm.

**Figure 16:**
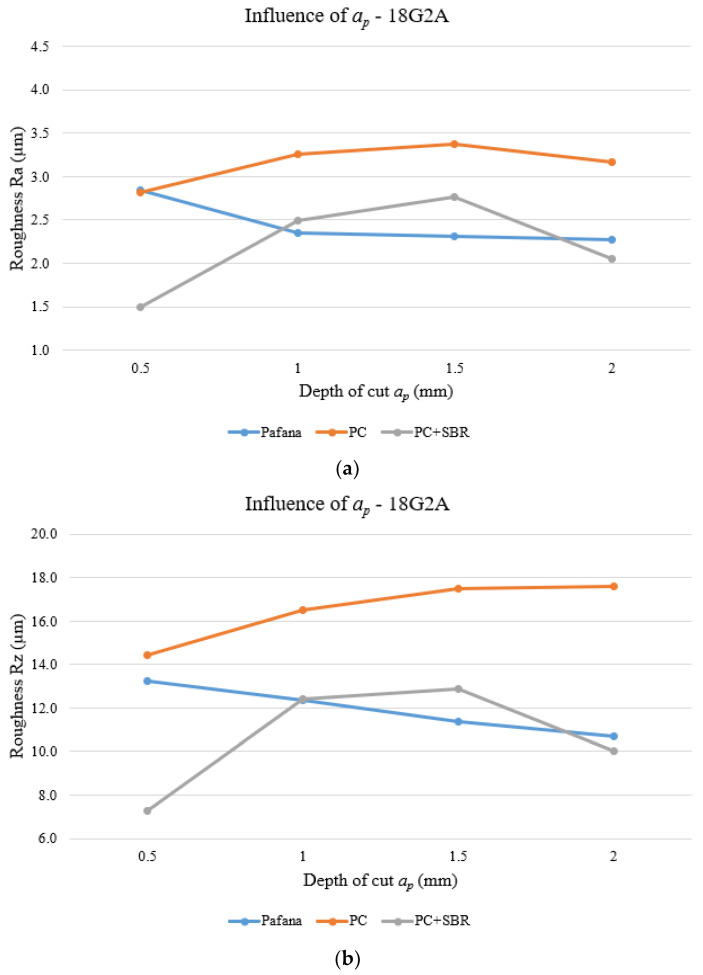
Effect of depth of cut on surface roughness for material 18G2A. (**a**) Ra, (**b**) Rz.

**Figure 17:**
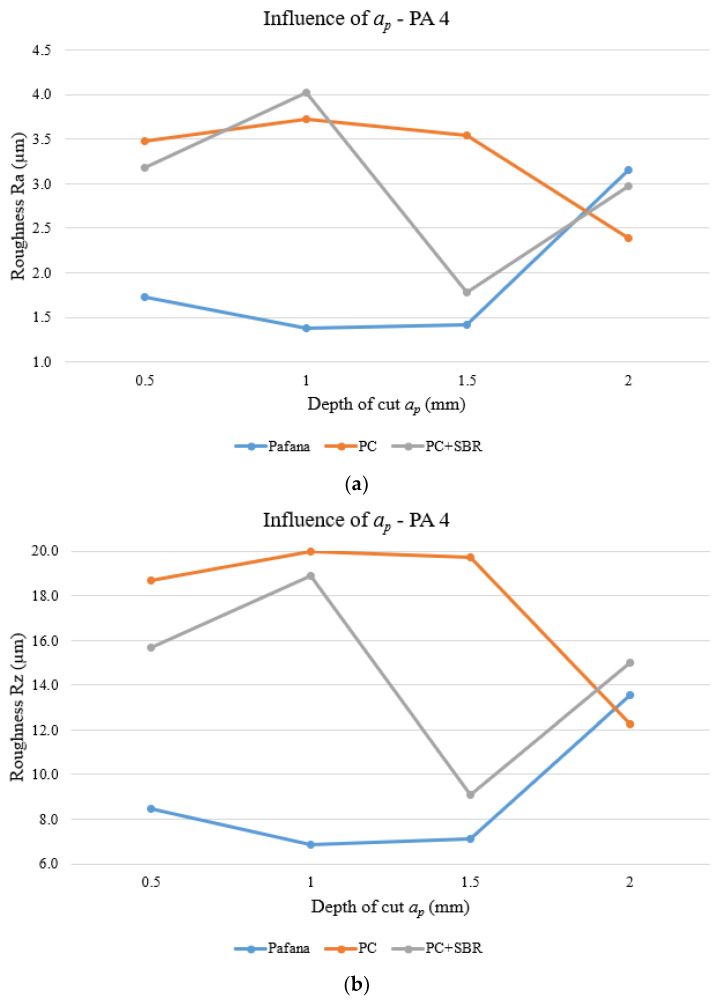
Effect of depth of cut on surface roughness for material PA4. (**a**) Ra, (**b**) Rz.

Figure 16 and Figure 17 show the results of testing the effect of depth of cut *a_p_* on surface roughness Ra and Rz for materials 18G2A and PA4 at constant rotational speed *n* = 710 rpm and constant feed rate *f* = 0.3 mm/rev.

As can be observed from the above figures for 18G2A steel, in the case of the PC boring bar, for both Ra and Rz roughness, there was a deterioration in the quality of the machined surface over the entire range studied in relation to PAFANA’s original tool. However, for the PC + SBR tool, it is possible to indicate the depths of cut (*a_p_* = 0.5 mm and *a_p_* = 2 mm) for which there was an improvement in the quality of the machined surface. Using a depth of cut setting from the middle of the tested range results in similar roughness values as for the original tool or a deterioration in surface quality.

As can be observed from the above figures for PA4 aluminum, in the case of the PC boring bar, for both Ra and Rz roughness, there was a significant deterioration in the quality of the machined surface in almost the entire range studied in relation to the original PAFANA tool. Only for depth of cut *a_p_* = 2 mm was a slight improvement in machined surface quality achieved. A similar behavior was noticed for the PC + SBR tool. In the case of roughness Ra, only setting the depth of cut at *a_p_* = 2 mm resulted in a slight improvement in the quality of the machined surface. On the other hand, in the case of roughness Rz, a deterioration in the quality of the machined surface was obtained over the entire range studied.

**Figure 18:**
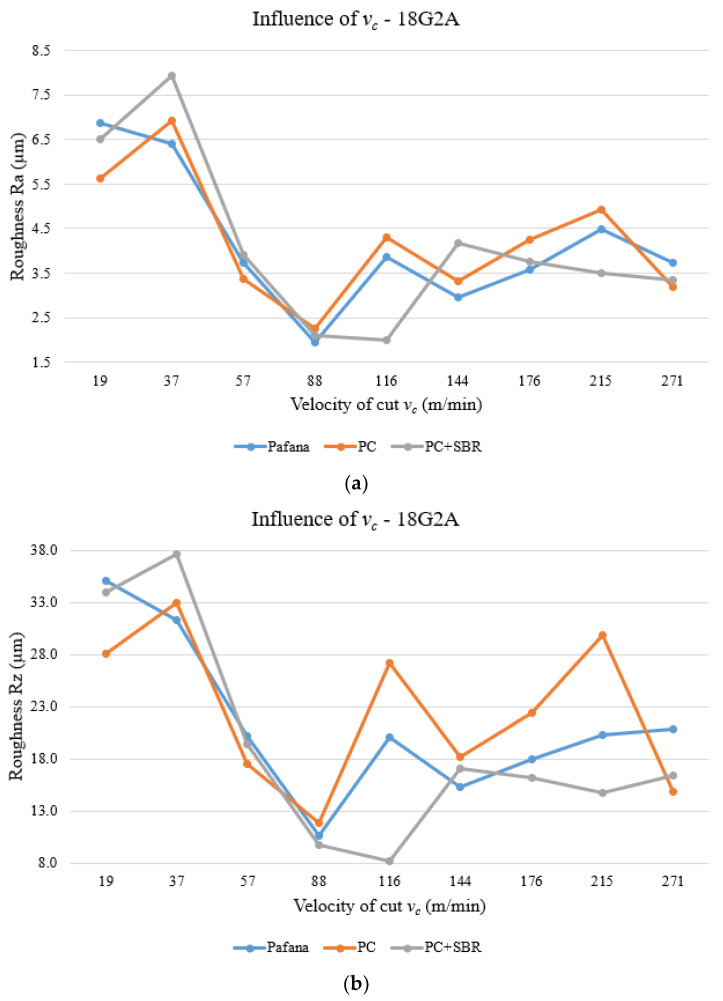
Effect of cutting speed on surface roughness for material 18G2A. (**a**) Ra, (**b**) Rz.

**Figure 19:**
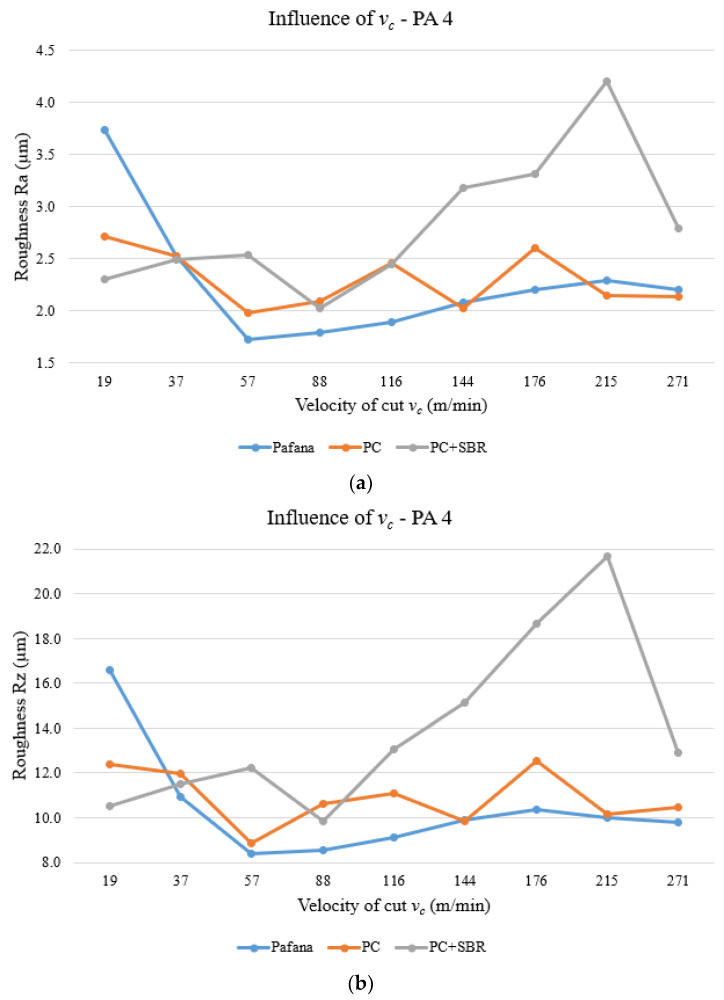
Effect of cutting speed on surface roughness for material PA4. (**a**) Ra, (**b**) Rz.

Figure 18 and Figure 19 show the results of testing the effect of cutting speed *v_c_* on surface roughness Ra and Rz for materials 18G2A and PA4 at constant depth of cut *a_p_* = 0.5 mm and constant feed rate *f* = 0.3 mm/rev.

As can be observed from the above figures, only at the beginning and end of the tested range of variable cutting speed *v_c_* is there an improvement in the quality of machined surface Ra and Rz for the 18G2A material. Outside of these settings, almost throughout the rest of the range the roughness values for all tools intermingle and are similar to each other. For the PC + SBR tool, for a cutting speed of *v_c_* = 116 m/min, an apparent improvement in machined surface quality can be seen as a decrease in the roughness values Ra and Rz.

For the machined PA4 material, there was a deterioration in the machined surface quality Ra and Rz practically over the entire range of variable cutting speed *v_c_* tested. Only the setting of the lowest cutting speed *v_c_* = 19 m/min resulted in an improvement in the quality of the machined surface.

**Figure 20:**
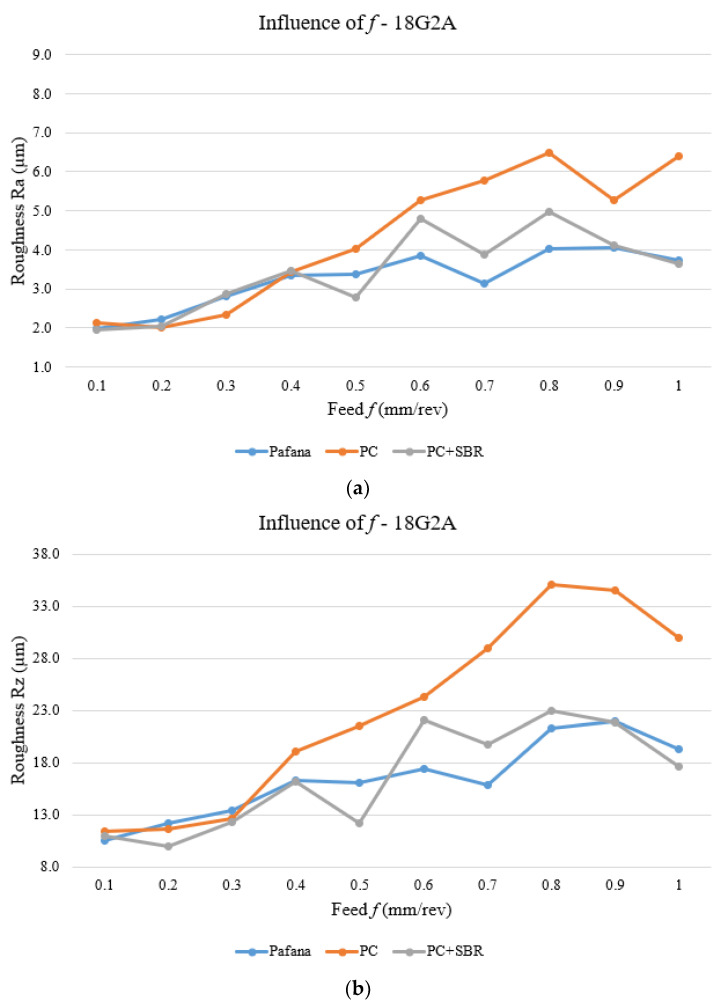
Effect of feed rate on surface roughness for material 18G2A. (**a**) Ra, (**b**) Rz.

**Figure 21:**
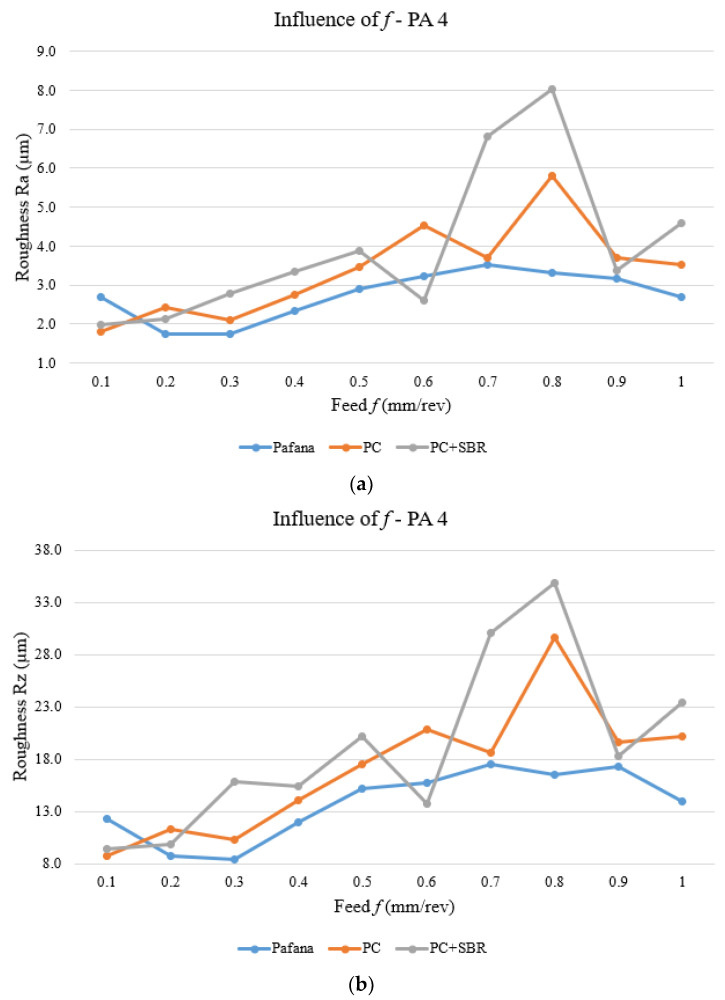
Effect of feed rate on surface roughness for material PA4. (**a**) Ra, (**b**) Rz.

Figure 20 and Figure 21 show the results of testing the effect of feed rate *f* on surface roughness Ra and Rz for materials 18G2A and PA4 at a constant depth of cut of *a_p_* = 0.5 mm and rotational speed *n* = 710 rpm.

As can be seen from the figures above, for the 18G2A material, there was a deterioration in the quality of the machined surface understood as an increase in the Ra and Rz parameters of the modified tools with respect to the original PAFANA boring bar in almost the entire range of the variable *f* studied. Only setting a low feed rate of *f* = 0.2 ÷ 0.3 mm/rev resulted in a slight improvement in the quality of the machined surface.

A similar situation to that of the 18G2A material is also presented in the figures of the PA4 material. With the use of modified tools, there was a deterioration in the surface roughness of the machined material. Only in the case for a value of feed *f* = 0.1 mm/rev did the quality of the machined surface improve, while in the entire remaining range the surface quality deteriorated, even drastically in places.

**Figure 22:**
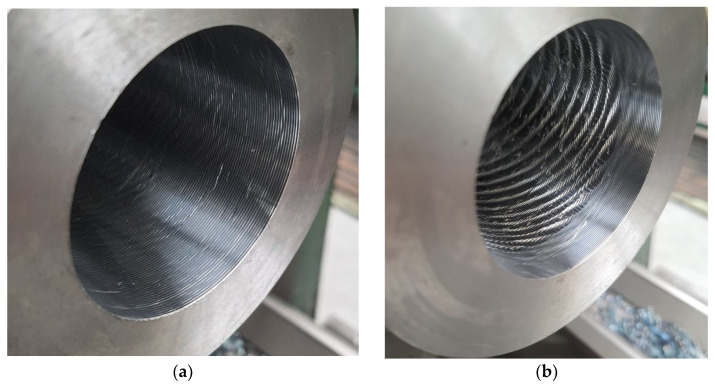
Example comparison of the appearance of the machined surface (**a**) using the original PAFANA tool and (**b**) using the PC prototype tool.

Figure 22 shows an example comparison of the appearance of the machined surface for the original PAFANA tool, as well as for the PC prototype tool. The comparison was made with the following boring parameters: *n* = 710 rpm, *f* = 0.8 mm/rev and *a_p_* = 0.5 mm. For the original tool, the following values of roughness parameters were obtained: Ra = 4.013 µm and Rz = 21.257 µm, while for the prototype tool these values were Ra = 6.476 µm and Rz = 35.051 µm, respectively.

From the comparison of the appearance of the machined surfaces, it can be concluded that when the hole was bored with the original PAFANA tool, normal rough boring without vibration took place, while when the hole was bored with the prototype PC tool, vibrations appeared, which negatively affected the appearance of the machined surface as well as the values of the roughness parameters Ra and Rz.

Poor surface finish and rapid tool wear are effects of chatter [26,27]. Chatter is a self-excited vibration caused by variation in chip thickness resulting from a time delay between the current cut and preceding cut. Chatter vibration in machining processes limits the accuracy and productivity of boring processes [26,28]. In order to achieve chatter-free long-bar boring, it is important to increase the static and dynamic stiffness of the boring bar. Static stiffness can be improved by optimizing bar geometry and using materials with a higher modulus of elasticity, and dynamic stiffness can be improved by increasing the damping of the structure [27,29].

What is more, other researchers have claimed that not only the mechanical properties of the boring bar depend on the limit of stability but also its fixation on the machine tool [3]. With an increase in cantilever length in particular, the mechanical properties of an inner core led to a considerably lower receptance at a higher natural frequency compared to the reference tool.

Chatter phenomena probably occur because of luck in the modification of machining parameters. Usually, when the tool is modified, new reasonable cutting parameters need to be selected to make these tool more effective than the original one [30]. In this study, due to use of not so stiff materials, the results of the modification were worse than the original PAFANA boring bar.

## 4. Summary and Conclusions

This ongoing project investigated the effect of modifying the design of a deep hole boring tool on dynamic and static properties. The research was divided into three stages: a numerical study, an experimental study and an operational study. The following conclusions can be drawn from the research:

- The numerical study showed that a suitable material for the core in the prototype boring bar is polymer concrete (PC) or polymer concrete combined with rubber (PC + SBR);
- The numerical study showed that a suitable design of the prototype tool would be a tool with a core size of *d* = 25 mm and *l* = 200 mm;
- The experimental study showed that for almost all characterized modes of vibration there was an increase in dynamic properties understood as a decrease in the amplitude of the transition function estimate and an increase in the free vibration damping coefficient for the prototype boring bar compared to the original boring bar;
- The operational tests showed that, in practically the whole tested range of cutting speed *v_c_*, the value of roughness of the machined surface Ra and Rz was lower for the original boring bar compared to the prototype boring bar;
- The operational tests showed that, in practically the whole examined range of feed rate *f*, the value of roughness of the machined surface Ra and Rz was lower for the original boring bar compared to the prototype boring bar;
- The operational tests showed that, in practically the whole studied range of depth of cut *a_p_*, the value of roughness of the machined surface Ra and Rz was lower for the original boring bar compared to the prototype boring bar.

Despite the promising results of both the numerical and experimental studies, the use of polymer concrete or polymer concrete doped with rubber granules (SBR) is not recommended as a core filling material for the shank section of deep hole boring tools. In-service testing has unequivocally shown that the quality of the machined surface is significantly inferior when machining with a prototype tool compared to the original tool, which, from a machining point of view, is crucial.

## Figures and Tables

**Table 1:** Values of variable parameters.

Type of Hole	D (mm)	L (mm)	d1 (mm)	d2 (mm)	d3 (mm)	l1 (mm)
Through hole	40	300	10, 20, 30	-	-	-
Bind hole	40	300	10, 20, 30	-	-	100, 200
Tapered hole	40	300	-	20, 30	10, 20	100, 200

**Table 2:** Comparison of static analysis results for different core materials.

Core Material	Displacement (mm)
Steel (PAFANA)	0.1466
Lead	0.1509
SBR	0.1628
PC	0.1252
PC + SBR	0.1395
Epoxy resin	0.1639

**Table 3:** Parameterization results for PC core.

		PC displacement (mm)					
		Length l1 (mm)					
		**50**	**100**	**150**	**200**	**250**	**283**
Diameter d1 (mm)	**10**	0.1423	0.1444	0.1404	0.1282	0.1299	0.1445
	**15**	0.1370	0.1445	0.1292	0.1233	0.1254	0.1476
	**20**	0.1347	0.1437	0.1266	0.1219	0.1243	0.1514
	**25**	0.1296	0.1428	0.1313	0.1252	0.1281	0.1613
	**30**	0.1260	0.1481	0.1423	0.1378	0.1400	0.1932
		PC mass (g)					
		Length l1 (mm)					
		**50**	**100**	**150**	**200**	**250**	**283**
Diameter d1 (mm)	**10**	3262	3231	3200	3169	3138	3118
	**15**	3247	3177	3108	3039	2969	2924
	**20**	3226	3103	2979	2856	2732	2651
	**25**	3199	3006	2813	2621	2428	2301
	**30**	3166	2889	2612	2335	2057	1874

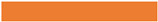
 The lowest value of displacement/mass of the boring bar. 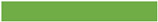 Most optimal combination indicated by Autodesk Inventor. 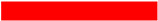 Displacement greater than original boring bar.

**Table 4:** Parameterization results for PC + SBR core.

		PC + SBR displacement (mm)					
		Length l1 (mm)					
		**50**	**100**	**150**	**200**	**250**	**283**
Diameter d1 (mm)	**10**	0.1433	0.1456	0.1477	0.1485	0.1491	0.1496
	**15**	0.1420	0.1435	0.1444	0.1473	0.1517	0.1506
	**20**	0.1417	0.1423	0.1431	0.1444	0.1552	0.1654
	**25**	0.1411	0.1418	0.1418	0.1421	0.1653	0.1744
	**30**	0.1405	0.1415	0.1453	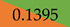	0.1853	0.2132
		PC + SBR mass (g)					
		Length l1 (mm)					
		**50**	**100**	**150**	**200**	**250**	**283**
Diameter d1 (mm)	**10**	3053	3033	3014	2995	2976	2956
	**15**	3032	2965	2905	2876	2752	2712
	**20**	3007	2891	2756	2674	2456	2321
	**25**	2981	2822	2680	2505	2251	2043
	**30**	2950	2751	2349	2225	1997	1733

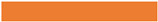
 The lowest value of displacement/mass of the boring bar. 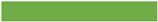 Most optimal combination indicated by Autodesk Inventor. 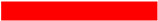 Displacement greater than original boring bar.

**Table 5:** Summary of test results in the vertical direction.

	PAFANA	PC	+/−	PC + SBR	+/−
**Mode 1**					
Frequency (Hz)	260.00	266.00	+2.3%	265.67	+2.2%
Amplitude ((m/s^2^)/N)	2.53	2.2	−13.0%	2.48	−2.0%
Damping ratio (-)	0.66	0.91	+37.9%	0.87	+31.8%
**Mode 2**					
Frequency (Hz)	1827.67	1777.00	−2.8%	-	-
Amplitude ((m/s^2^)/N)	4.22	1.00	−76.3%	-	-
Damping ratio (-)	0.63	1.02	+61.9%	-	-
**Mode 3**					
Frequency (Hz)	2652.67	-	-	-	-
Amplitude ((m/s^2^)/N)	1.62	-	-	-	-
Damping ratio (-)	0.27	-	-	-	-

**Table 6:** Summary of test results in the horizontal direction.

	PAFANA	PC	+/−	PC + SBR	+/−
**Mode 1**					
Frequency (Hz)	314.33	343.67	−2.2%	345.33	+1.2%
Amplitude ((m/s^2^)/N)	1.75	2.04	+16.6%	2.05	+17.1%
Damping ratio (-)	1.78	1.67	−6.2%	1.65	−7.3%
**Mode 2**					
Frequency (Hz)	1761.00	1721.00	−2.2%	-	-
Amplitude ((m/s^2^)/N)	3.45	1.61	−53.3%	-	-
Damping ratio (-)	0.82	1.58	+92.7%	-	-
**Mode 3**					
Frequency (Hz)	2654.00	-	-	-	-
Amplitude ((m/s^2^)/N)	1.24	-	-	-	-
Damping ratio (-)	0.28	-	-	-	-

**Table 7:** Results of measurements of displacements of the boring bar and table frame under static loads for the PAFANA boring bar.

**Top sensor**									
Measurement	Mass 1			Mass 2			Mass 3		
	Before (µm)	In the process (µm)	Difference (µm)	Before (µm)	In the process (µm)	Difference (µm)	Before (µm)	In the process (µm)	Difference (µm)
1	1294	1105	189	1307	901	406	1306	699	607
2	1298	1109	189	1293	899	394	1304	701	603
3	1298	1102	196	1294	902	392	1312	703	609
**Bottom sensor**									
Measurement	Mass 1			Mass 2			Mass 3		
	Before (µm)	In the process (µm)	Difference (µm)	Before (µm)	In the process (µm)	Difference (µm)	Before (µm)	In the process (µm)	Difference (µm)
1	5	79	74	2	178	176	9	269	260
2	3	78	75	14	178	165	18	272	254
3	1	80	79	14	181	167	20	273	253
**The average value of the differences in the top and bottom sensor readings** **(µm)**			**115**	**The average value of the differences in the top and bottom sensor readings** **(µm)**		**228**	**The average value of the differences in the top and bottom sensor readings** **(µm)**		**351**

**Table 8:** Results of measurements of displacements of the boring bar and table frame under static loads for the PC boring bar.

**Top sensor**									
Measurement	Mass 1			Mass 2			Mass 3		
	Before (µm)	In the process (µm)	Difference (µm)	Before (µm)	In the process (µm)	Difference (µm)	Before (µm)	In the process (µm)	Difference (µm)
1	1211	1017	194	1264	851	413	1205	587	618
2	1245	1045	200	1233	819	414	1211	590	621
3	1235	1032	203	1221	813	408	1204	589	615
**Bottom sensor**									
Measurement	Mass 1			Mass 2			Mass 3		
	Before (µm)	In the process (µm)	Difference (µm)	Before (µm)	In the process (µm)	Difference (µm)	Before (µm)	In the process (µm)	Difference (µm)
1	5	82	77	6	175	169	1	261	260
2	2	74	72	0	161	161	12	262	250
3	2	76	74	1	164	163	13	261	248
**The average value of the differences in the top and bottom sensor readings** **(µm)**			**125**	**The average value of the differences in the top and bottom sensor readings** **(µm)**		**247**	**The average value of the differences in the top and bottom sensor readings** **(µm)**		**365**

**Table 9:** Results of measurements of displacements of the boring bar and table frame under static loads for the PC + SBR boring bar.

**Top sensor**									
Measurement	Mass 1			Mass 2			Mass 3		
	Before (µm)	In the process (µm)	Difference (µm)	Before (µm)	In the process (µm)	Difference (µm)	Before (µm)	In the process (µm)	Difference (µm)
1	1320	1117	203	1321	914	407	1328	692	636
2	1315	1119	196	1311	913	398	1310	697	613
3	1311	1109	202	1310	905	405	1309	701	608
**Bottom sensor**									
Measurement	Mass 1			Mass 2			Mass 3		
	Before (µm)	In the process (µm)	Difference (µm)	Before (µm)	In the process (µm)	Difference (µm)	Before (µm)	In the process (µm)	Difference (µm)
1	10	87	77	10	173	163	4	261	257
2	14	87	73	15	171	156	16	262	246
3	15	88	73	16	177	161	16	261	245
**The average value of the differences in the top and bottom sensor readings** **(µm)**			**126**	**The average value of the differences in the top and bottom sensor readings** **(µm)**		**243**	**The average value of the differences in the top and bottom sensor readings** **(µm)**		**370**

## Data Availability

Data are contained within the article.
